# Evaluating Current Practice of Prescribing as Required Medications for Psychiatric Inpatients

**DOI:** 10.1192/bjo.2024.486

**Published:** 2024-08-01

**Authors:** Naveen Lalli, Olabisitaiye Abu, Safyan Tariq, Vijay Perla, Nilamadhab Kar

**Affiliations:** Penn Psychiatric Hospital (NHS), Wolverhampton, United Kingdom

## Abstract

**Aims:**

*Pro re nata* (PRN) medications are commonly prescribed for psychiatric patients on admission, often at maximum daily dose (MaxD). We intended to evaluate prescribing patterns for PRN medications, their MaxD, and rationale, specifically in the first seven days in the hospital, along with any concerns of associated physical illnesses.

**Methods:**

All the inpatients on a specific date, admitted to adult and old age wards of a general psychiatric hospital, for at least 7 days, were recruited for this service evaluation. Data regarding the prescribing of promethazine, lorazepam, zopiclone as PRN, patient demographics, and psychiatric and physical diagnoses were collected using inpatient drug cards and electronic patient notes.

**Results:**

Out of 52 inpatients, 14 were excluded (4 admitted for < 7 days, and 10 had missing data), leading to a sample size of 38 patients. On admission, a considerable proportion of patients were prescribed promethazine (82%), lorazepam (76%), and zopiclone (50%). More than half (63%) of patients on promethazine were started on 100 mg MaxD, of which 13% had reasons for prescription, and 33% had reasons for the MaxD were noted. None of the old-age patients was prescribed 100 mg of promethazine. During first 7 days, patients used on average 15%, 14% and 29% of the total prescribed dose of PRN promethazine, lorazepam and zopiclone; and 35%, 45% and 47% of patients did not use any PRN drugs. Only one patient used 100% of the available PRN lorazepam and zopiclone. Patients with current illicit substance misuse, used PRN slightly more frequently; promethazine (16% v 12%), lorazepam (20% v 14%) and zopiclone (46% v 24%) compared with those with no misuse. With a current risk of aggression or agitation, all female patients were prescribed PRN promethazine or lorazepam, compared with 86% of male patients.

In regards to British National Formulary (BNF) cautions of associated physical illness, one patient with glaucoma, and one epilepsy was prescribed promethazine; three patients with respiratory condition were prescribed PRN lorazepam; and six patients with depression and four with current drug user were prescribed PRN zopiclone.

Considering diagnoses, promethazine, lorazepam and zopiclone were used by varying proportions of the patients: schizophrenia (10%, 3%, 0%), bipolar affective disorder (0%, 14%, 57%), depression (27%, 11%, 38%), personality disorder (15%, 28%, 48%) respectively.

**Conclusion:**

Psychiatric inpatients were prescribed MaxD of PRN medications more than what is being administered. Documentation of rationale for prescribing PRN medications and dose is needed.

